# Suitable Cottonseed Protein Concentrate Supplementation in Common Carp (*Cyprinus carpio*) Serves as an Effective Strategy for Fish Meal Sparing Based on Improvement in Intestinal Antioxidant Capacity, Barrier and Microbiota Composition

**DOI:** 10.3390/antiox13040436

**Published:** 2024-04-04

**Authors:** Ze Fan, Kaibo Ge, Di Wu, Liansheng Wang, Jinnan Li, Chenhui Li, Meng Zhou, Haitao Zhang, Linghong Miao, Xianping Ge

**Affiliations:** 1 Key Laboratory of Aquatic Animal Diseases and Immune Technology of Heilongjiang Province, Heilongjiang River Fisheries Research Institute, Chinese Academy of Fishery Sciences, Harbin 150070, China; fanze@hrfri.ac.cn (Z.F.); gekaibo@hrfri.ac.cn (K.G.); wudi@hrfri.ac.cn (D.W.); lijinnan@hrfri.ac.cn (J.L.); lichenhui@hrfri.ac.cn (C.L.); 2 Innovative Institute of Animal Healthy Breeding, College of Animal Sciences and Technology, Zhongkai University of Agriculture and Engineering, Guangzhou 510225, China; 3 Key Laboratory of Aquatic, Livestock and Poultry Feed Science and Technology in South China, Ministry of Agriculture and Rural Affairs, Guangdong Evergreen Feed Industry Co., Ltd., Zhanjiang 524000, China; lh0888@163.com; 4 Key Laboratory of Fresh Water Fisheries and Germplasm Resources Utilization, Ministry of Agriculture and Rural Affairs, Freshwater Fisheries Research Center, Chinese Academy of Fishery Sciences, Wuxi 214081, China; miaolinghong@ffrc.cn (L.M.); gexp@ffrc.cn (X.G.)

**Keywords:** common carp (*Cyprinus carpio*), fishmeal replacement, cottonseed protein concentrate (CPC), intestinal antioxidant capacity, intestinal immune barrier

## Abstract

The application of cottonseed protein concentrate (CPC) is an effective strategy to moderate the shortage of fish meal (FM) for the aquafeed industry. However, little attention has been paid to the effects of replacing fishmeal with CPC on cyprinid fish. This study used common carp (*Cyprinus carpio*) as the biological model and assessed the potential of applying CPC as a substitute for fishmeal in the diet of common carp. The proportion of fish meal substituted with CPC in the six diets was 0% (CPC0), 25% (CPC25), 50% (CPC50), 75% (CPC75), and 100% (CPC100). Each diet was fed to three replicate groups of common carp (4.17 ± 0.02 g) for 56 days. Results revealed that the CPC50 group significantly increased the growth indexes via up-regulating the genes of the GH/IGF axis and the TOR pathway. The intestinal digestive ability was also elevated in the CPC50 group via markedly increasing intestinal villus height, protease and lipase activities in the whole intestine, and the amylase activity of the foregut and midgut. The CPC50 group captured significantly higher activities and gene expressions of antioxidant enzymes and lower malonaldehyde contents via evoking the Nrf2/Keap1 signal pathway. The CPC50 group enhance the intestinal mechanical barrier via up-regulating the gene expressions of tight junction proteins and heighten the intestinal biological barrier by increasing the probiotics (*Lactococcus*) and decreasing the harmful bacteria (*Enterococcus*). But excessive substitution levels (75% and 100%) would compromise growth performance, intestinal antioxidant capacity, and immune function. The optimum substitution level was estimated to be 46.47%, 47.72%, and 46.43% using broken-line regression analyses based on mass gain rate, protein efficiency ratio, and feed conversion rate. Overall, the fishmeal in common carp feed could be substituted up to 50% by CPC without negative influence on growth, feed utilization, and or intestinal health.

## 1. Introduction

Common carp (*Cyprinus carpio*) are widely cultivated throughout the freshwater culture areas on account of their strong adaptability, low oxygen tolerance, rapid growth, and easy artificial propagation [1]. Common carp is not only a freshwater fish with high economic value [2], but also has significant research value in the field of aquatic ecosystem science [3,4]. Based on statistics released by the Food and Agriculture Organization of the United Nations (FAO) in 2022, the total global production of common carp has attained a staggering 4.26 million tons, accounting for 8.6% of finfish production in inland aquaculture (FAO, 2022) [4]. Common carp are rarely involved in international trade, but they affect the nutritional intake of millions of people [4]. It is foreseeable that the high production of common carp brings about a high demand for feed, especially the demand for increasingly precious fishmeal resources. Although 6–8% fishmeal provision in common carp feed could be generally inferior to feed of carnivorous fish [5,6], the shortage and costliness of fishmeal have been still perceived as the key factors restricting the development of the common carp aquaculture industry in the future [7,8]. Hence, it is urgent and pivotal to find appropriate ways to achieve the goal of fish meal reduction to satisfy the sustainable development of the common carp industry, even for the cyprinid fish farming industry [9].

In recent years, the development of novel alternative protein sources has become one of the most effective ways to deal with the above dilemmas [10]. According to the current research on common carp, the involved novel protein sources mainly focus on animal protein sources, including fermented Antarctic krill meal [11], chicken intestinal hydrolysates [12], and Crayfish Shell Meal [13], whereas there is a relative lack of research on the replacement of fish meal with novel plant protein sources. Among the multitudinous novel plant protein sources, cottonseed protein concentrate (CPC) has become a research hotspot or focus of aquafeed [14]. CPC is made from common cottonseed meal with low gossypol, of which the potential to replace fish meal has been demonstrated in the feed for carnivorous fish such as golden pompano (*Trachinotus ovatus*) [15], largemouth bass (*Micropterus salmoides*) [16], and red drum (*Sciaenops ocellatus*) [17]. The reason why CPC can replace fishmeal at a high level could be that CPC has a higher nutritional value of protein, better palatability, and contains fewer allergenic antigens by way of low-temperature extraction, dephenolization, and desugarization [14]. For representative cyprinid fish, the studies of grass carp (*Ctenopharyngodon idellus*) [18,19] have also confirmed the feasibility of replacing soybean meal in feed with cottonseed protein concentrate. Our previous study also found that substituting soybean meal for CPC can not only promote the growth of common carp and feed utilization efficiency but also optimize the amino acid composition, texture characteristics, and water-holding capacity of muscle and enhance the flavor and taste of fish [20]. However, little attention has been paid to the effects of replacing fishmeal with CPC on common carp or other cyprinid fish.

How to confirm that a novel animal or plant protein source can effectively replace fish meal is a key issue that must be paid attention to. In addition to extensive concerns about fish growth and the nutritional composition of fish or feed ingredients, more and more attention has been paid to the intestinal health of fish, both in the study of nutritional regulation [21] and environmental stress [22]. Especially for common carp as a representative of cyprinid fish, the intestine has been viewed as a crucial tissue integrating digestive, immune, and metabolic functions [23]. It has been reported that the substitution of fishmeal with fermented soybean meal in *Carassius auratus* feeds could increase the width and length of intestinal villi to expand intestinal absorption area, thereby improving intestinal absorption ability [24]. Besides, Lu et al. [25] observed that the replacement of dietary soybean meal with defatted black soldier fly larvae meal of less than 50% could improve the intestinal antioxidant capacity of grass carp, whereas replacement levels above 50% would induce histopathological damage to intestinal tissues. In our previous study, the fishmeal in common carp feed could be substituted up to 50% by chicken intestinal hydrolysates without negative influence on intestinal antioxidant activity and barrier function [12]. By far, the potential impacts of replacing fishmeal with CPC on the intestine health of cyprinid fish have been lacking.

For the status quo outlined above, the aim of the current study was to assess the effects of CPC replacing dietary fishmeal on the growth performance, intestinal antioxidant capacity, barrier, and microbiota composition of common carp. Research achievement provides essential data for the application of CPC into the commercial diet for common carp, even for omnivorous cyprinid fish, and also lays a practical foundation for fish meal reduction action.

## 2. Materials and Methods

### 2.1. Design and Production Process of Experimental Diets

CPC was purchased from Xinjiang Jinlan Plant Protein Co., Ltd., Shihezi City, China, which contains 63.51% crude protein, 1.80% crude lipid, 0.85% methionine, and 2.47% lysine. In the current research, CPC was used at concentrations of 25 g/kg, 50 g/kg, 75 g/kg, and 100 g/kg to replace dietary fish meal at levels of 25% (CPC25), 50% (CPC50), 75% (CPC75), and 100% (CPC100). A basal diet containing 10% fish meal was regarded as the control group (CPC0). The experimental diets were designed to be isonitrogenous and isolipidic as displayed in Table 1. The Feed production process agreed with the consensus from previous studies [7]. The dietary pellets with a diameter of 2 mm were toasted in a drying closet at 60 °C for 30 min, and then naturally air dried. The air-dried dietary pellets were preserved in a freezer at −20 °C for feeding trials.

### 2.2. Design and Management of the Feeding Trial

The experiment was conducted at the water recirculating aquaculture facilities of the Fish Nutrition and Feed Department in the Heilongjiang River Fisheries Research Institute (Harbin, China). Healthy juvenile common carp were purchased from the commercial farm (Chengdu, China) and temporarily reared in 0.5 m^3^ aquariums and fed commercial diets (300 g/kg crude protein, 60 g/kg crude lipid, Guangzhou Haid Group Co., Ltd., Guangzhou, China) during the 2-week acclimatization period. At the beginning of the experiment, the circulating water system was adequately disinfected with potassium permanganate. After 24-h fasting, a total of 375 healthy common carp with an approximate size of 4.17 ± 0.02 g of mean wet mass were randomly allotted into 5 groups with 3 replicates for the feeding trial. Fish were cultured in 15 aquariums (0.5 m^3^) and fed three times daily (08:00, 13:00, and 17:00) by the standard of a ratio of 7% to 8% of the body mass [26]. The experimental water underwent aeration treatment and was replaced daily at a rate of 30%. During the 56-d course of the feed trial, the water temperature, total ammonia, and dissolved oxygen were maintained at 24–26 °C, about 0.20 mg/L and 5.8–6.2 mg/L, respectively.

### 2.3. Sample Acquisition and Analysis Methods

#### 2.3.1. Calculation of Growth and Feed Utilization

**Figure 1:**
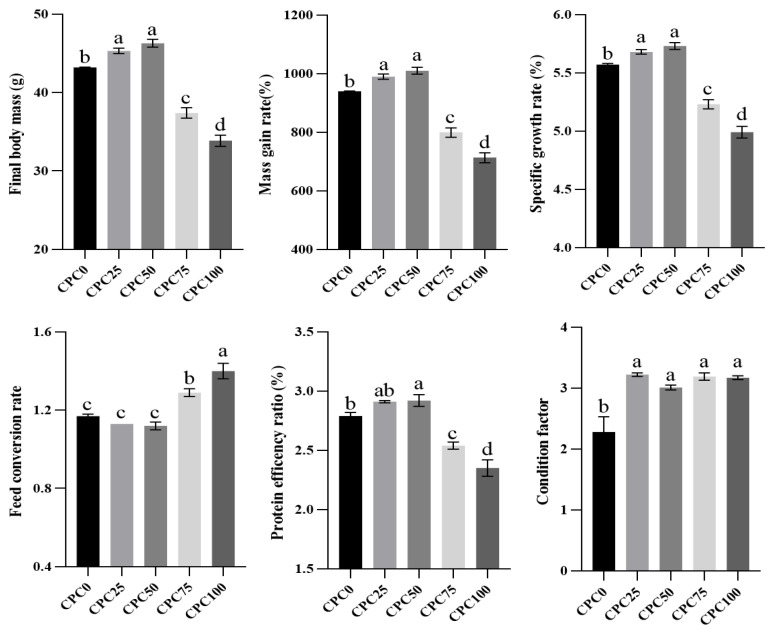
Parameters of growth and feed utilization of common carp-fed diets with fish meal substituted by CPC. Values were presented as mean ± SE. Bars with different letters indicated significant differences (*p* < 0.05). Note: Mass gain rate (MGR, (%) = 100 × [(final mass − initial mass)]/initial mass; Specific growth rate (SGR, %/d) = 100 × [(Ln final mass − Ln initial mass)/56 days]; Feed conversion rate (FCR) = [100 × total feed intake/(final mass − initial mass)]; Protein efficiency ratio (PER, %) = 100% × [final mass (g) − initial mass (g))/(total feed intake (g) × content of dietary protein (%)]; Condition factor (CF, g/cm^3^) = mass (g)/length^3^ (cm).

At the end of the 56-day feeding trial, 24-h fasting was carried out before measurement and sample acquisition. For accurate calculation of survival rate (SR), mass gain rate (MGR), and specific growth rate (SGR), common carp in each aquarium were counted and weighed, respectively. As for feed conversion ratio (FCR) and protein efficiency ratio (PER), total feed intake was accurately documented during the feeding trial. Randomly collected six fish from each aquarium were used to measure body length and weight to calculate the condition factor (CF). The formulas for calculating the above data are listed in the notes of Figure 1.

#### 2.3.2. Detection of Serum Biochemical Indexes

Six randomly selected fish from each aquarium were selected to draw blood from the caudal vein. The collected blood was kept at 4 °C for 12 h and then centrifuged at 4 °C with 1062× *g* for 15 min to acquire serum. The serum detection experiment was conducted with a microplate reader (Thermo Multiskan GO 1510, Thermo Fisher Scientific (China) Co., Ltd., Shanghai, China) depending on kits manufactured by Nanjing Jiancheng Institute of Biological Engineering, Nanjing, in which specific detection indexes included total protein (TP), blood urea nitrogen (BUN), triglyceride (TG), total cholesterol (TC), high-density lipoprotein cholesterol (HDL-C), low-density lipoprotein cholesterol (LDL-C), aspartate transaminase (AST), and alanine aminotransferase (ALT) (Cat No. in order: A045-4-2, A110-1-1, A111-1-, A112-1-, A113-1-1, C010-2-1, C009-2-1).

#### 2.3.3. Analysis of the Intestinal Activities of Digestion and Antioxidant Enzymes

Three randomly selected fish were used to collect the intestine samples for subsequent enzyme activity detection from each aquarium. After quick freezing in liquid nitrogen, all the intestine samples were subsequently preserved in a −80 °C refrigerator. Referring to the kits (Shanghai EnzymeLink Ltd., Shanghai, China) instructions to extract the intestinal supernatant, the activity determination of protease and superoxide dismutase (SOD) was carried out via Enzyme-linked immunosorbent (ELISA) kits (Bioroyee (Beijing) Biotechnology Co., Ltd., Beijing, China), which adopt the double antibody sandwich method to measure the activity of the enzyme in tissue-related liquid samples. The activities or contents of lipase (A054-1-1), amylase (C016-1-1), catalase (CAT, A007-2-1), glutathione (GSH, A006-2-1), glutathione peroxidase (GSHPx, A005-1-2), malonaldehyde (MDA, A003-1-2), and total protein (A045-2-2) in the intestinal homogenates were detected using kits produced by Nanjing Jiancheng Bioengineering Institute.

#### 2.3.4. Intestinal mRNA Expression Analysis

The mRNA levels of genes-related to the growth axis, protein synthesis, antioxidation, immune barrier, and cytokines in the intestine were measured as described previously [7]. Briefly, the RNAiso method is utilized to isolate mRNA from the intestine samples. Subsequently, cDNA was acquired through reverse transcription of a 1-μg mRNA, thereby executing subsequent qPCR detection. The applied primer sequences and serial numbers of target genes and the reference gene (β-actin) in this study are listed in Table 2. The amplification efficiency range of primers in our study was maintained to be 95–110%. The 2^−ΔΔCt^ method was applied to calculate the relative mRNA abundance of the target genes [27].

#### 2.3.5. Histological Observation of the Intestine

Around 0.5 cm of fresh intestine sample was dissected from farmed common carp, gently rinsed to remove the intestinal mucosa and contents adhering to the tissue, and then fixed in 4% paraformaldehyde fixative. The preparation process for intestinal slices was based on the previous methods in our laboratory [7]. Then, the sections were observed on a MD 2000B Leica light microscope produced by Leica Microsystems (Shanghai) Trading Co., Ltd., Shanghai, China.

#### 2.3.6. Intestinal Microbiota Analysis Procedure

The detection of intestinal microbiota was consigned to Shanghai Majorbio Bio-pharm Technology Co., Ltd. Shanghai, China, and the specific operation process was as described in previously reported protocols [28]. Total microbiota genomic DNA was separated from the cecum contents of common carp by virtue of the E.Z.N.A. DNA Kit (Omega Bio-tek, Norcross, GA, USA). The V3-V4 variable regions of the bacterial 16S rRNA gene were amplified depending on degenerate PCR primers 338F/806R through an ABI GeneAmp 9700 PCR thermocycler (ABI, Los Angeles, CA, USA). Next, an Illumina MiSeq PE300 platform (Illumina (China) SCIENTIFIC Equipment Co., Ltd., Shanghai, China) was applied to paired-end sequencing of purified amplicons.

### 2.4. Design and Preparation of Experimental Diets

The difference in the different alternative-level treatments was analyzed by one-way analysis of variance (ANOVA) and Duncan’s test via SPSS 23.0 software. The data were presented as the mean ± standard error (SE), and a column diagram was drawn via GraphPad Prism 7.0 software; meanwhile, broken-line regression analyses were conducted to investigate the optimum replacement level based on MGR, FCR, and PER [7]. Statistically significant differences among the detected data were labeled as *p* < 0.05, except as otherwise noted.

## 3. Results

### 3.1. Parameters of Growth and Feed Utilization

**Figure 2:**
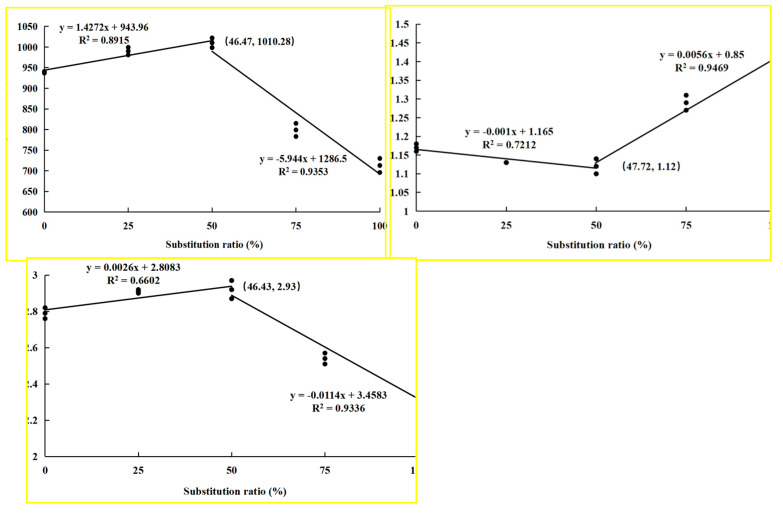
Broken-line regression analysis of mass gain rate (%), feed conversion rate, and protein efficiency rate for dietary fish meal substituted by CPC.

No mortality occurred in all groups across the feeding period. Growth parameters such as final body mass, MGR, SGR, and CF were evaluated. The CPC25 and CPC50 groups showed a significantly positive effect on final body mass, MGR, and SGR in comparison with the CPC0 group (*p* < 0.05), whereas the CPC75 and CPC100 groups showed a significantly negative effect. With regard to CF, all CPC replacement groups obtained significantly higher values than the CPC0 group (*p* < 0.05). Feed utilization parameters, including FCR and PER, were calculated. The markedly lower FCR was observed in the CPC50 group compared to the CPC75 and CPC100 groups (*p* < 0.05); consequently, a markedly higher PER was also evident during the feeding period for the CPC50 group than the CPC0, CPC75, and CPC100 groups (*p* < 0.05) (Figure 1). Based upon the broken-line regression analysis of MGR, PER, and FCR against CPC replacement levels, the optimal CPC replacement levels were, respectively, 46.47%, 47.72%, and 46.43% of the feed (Figure 2).

### 3.2. Biochemical Components of the Serum

Table 3 showed that the contents of TP, ALB, and HDL-C initially increased and then decreased, and the CPC50 group showed significantly higher TP content than the CPC0 and CPC100 groups, and significantly higher HDL-C content than the other groups (*p* < 0.05). On the contrary, the contents of BUN, TG, and LDL-C showed an opposite tendency, and their minimum values were found in the CPC50 group. Specifically, the CPC50 group possessed lower BUN and HDL-C contents than the other groups except the CPC25 group and lower LDL-C contents than the CPC100 group (*p* < 0.05). In addition, compared with the other groups except CPC50, the ALT in the CPC75 group was significantly higher (*p* < 0.05). Compared with the other groups, the AST in the CPC100 group was significantly higher (*p* < 0.05).

### 3.3. Intestinal Digestive Enzyme Activities

**Figure 3:**
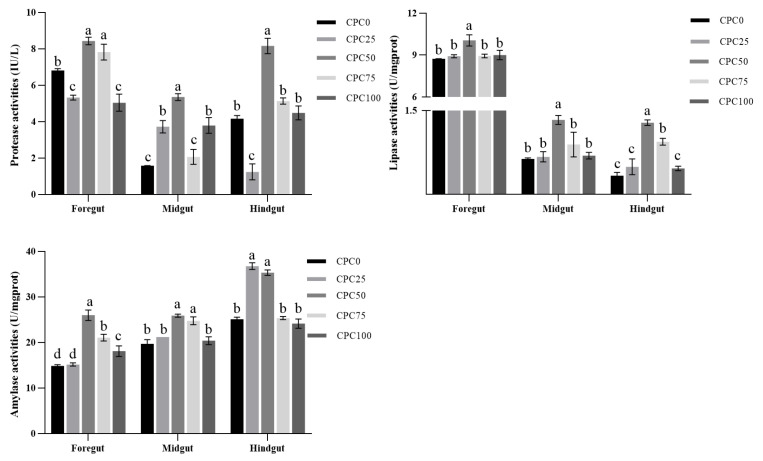
Intestinal digestive enzyme activities of common carp-fed diets with fish meal substituted by CPC. Values were presented as mean ± SE. Bars with different letters indicated significant differences (*p* < 0.05).

The replacement of fishmeal with CPC affected the digestive enzyme activities of the foregut, midgut, and hindgut. After CPC replacement, although the protease activities remained a noteless tendency in the whole intestine, the CPC50 group was still observed to display significantly higher values in three intestinal segments than other groups (*p* < 0.05). On the contrary, the intestinal lipase and amylase activities were elevated in CPC replacement groups, this being more evident in the lipase activities of three intestinal segments and the amylase activities of the foregut and midgut. Among these, the CPC50 group exhibited markedly higher lipase and amylase activities (*p* < 0.05), followed by the CPC75 group (Figure 3).

### 3.4. Intestinal Antioxidant Indexes Evaluation

**Figure 4:**
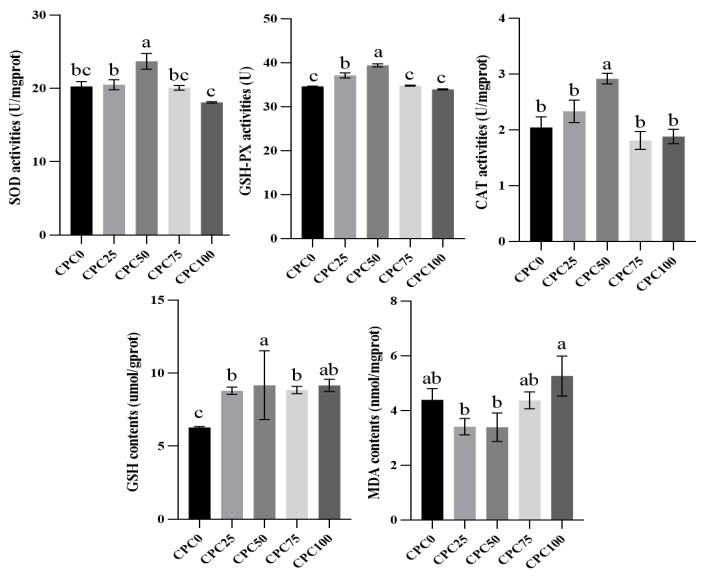
Intestinal antioxidant indexes of common carp-fed diets with fish meal substituted by CPC. Values were presented as mean ± SE. Bars with different letters indicated significant differences (*p* < 0.05).

As shown in Figure 4, 25% and 50% replacement of fish meal with CPC triggered the increments of SOD, CAT, and GSHPx activities in the intestines. Specifically, markedly higher activities of three enzymes were found in the CPC50 group. CPC replacement brought higher intestinal GSH contents, and the CPC50 group possessed significantly higher GSH contents than the other groups (*p* < 0.05). Regarding the MDA contents, significant reductions in the CPC25 and CPC50 groups were observed compared to the CPC100 group (*p* < 0.05).

### 3.5. Intestinal Relative Expressions of Growth Axis and Protein Synthesis Genes

**Figure 5:**
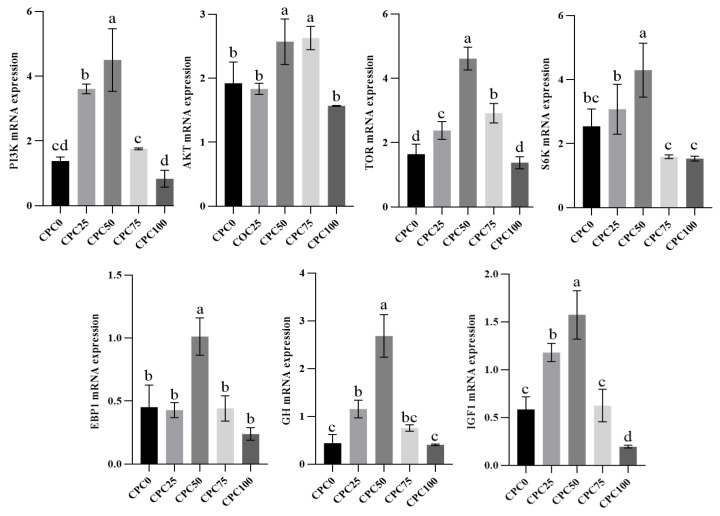
Intestinal relative expressions of growth axis and protein synthesis genes of common carp fed diets with fish meal substituted by CPC. Values were presented as mean ± SD. Bars with different letters indicated significant differences (*p* < 0.05).

In the intestine, the relative expressions of *GH*, *IGF1*, *PI3K*, *AKT*, *TOR*, *S6K*, and *4ebp1* in each group first increased and then decreased, reaching the highest levels in the CPC50 group. For the growth axis genes, the *GH* and *IGF1* relative expressions in the CPC50 group were significantly up-regulated compared to the other groups (*p* < 0.05). For the protein synthesis genes, the relative expressions of *PI3K*, *TOR*, *S6K*, and *4ebp1* in the CPC50 group were markedly higher than the other groups (*p* < 0.05). Meanwhile, it also needs to be mentioned that all the relative expressions of growth axis and protein synthesis genes reached the lowest levels in the CPC100 group. Of these, the relative expressions of *IGF1*, *PI3K*, and *TOR* in the CPC100 group were markedly down-regulated compared to the CPC25, CPC50, and CPC75 groups (*p* < 0.05) (Figure 5).

### 3.6. Intestinal Relative Expressions of Antioxidation Genes

**Figure 6:**
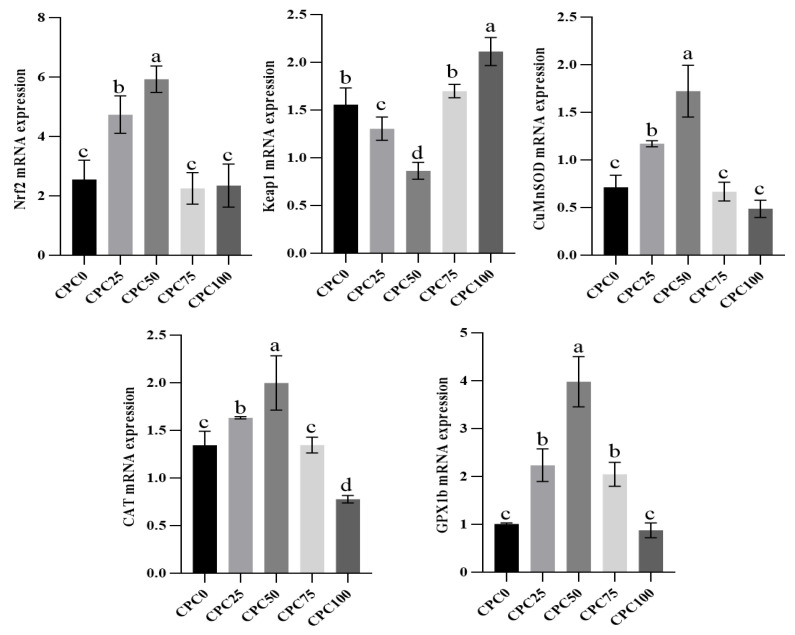
Intestinal relative expressions in antioxidation genes of common carp-fed diets with fish meal substituted by CPC. Values were presented as mean ± SE. Bars with different letters indicated significant differences (*p* < 0.05).

As the key actor involved in antioxidation, we studied the genes coding for *Nrf2*, *Keap1*, *CuZnSOD*, *CAT*, and the *GPx1b* protein. Significant differences in the expression of studied genes were found among five diets (*p* < 0.05). The relative expression levels of *Nrf2*, *CuZnSOD*, *CAT*, and *GPx1b* were significantly elevated in the CPC50 group (*p* < 0.05). Conversely, the relative expression levels of *Keap1* in the CPC50 group were significantly decreased (*p* < 0.05). Noteworthy, the CPC100 group obtained the lowest expression levels of *Nrf2*, *CuZnSOD*, *CAT*, and *GPx1b* and the highest Keap1 expression level. Of these, the CAT expression level in the CPC100 group was significantly lower than the other group (*p* < 0.05), whereas the *Keap1* expression level in the CPC100 group was significantly higher than the other group (*p* < 0.05) (Figure 6).

### 3.7. Intestinal Relative Expressions of Immune Barrier and Cytokines Genes

With the rising replacement level of fishmeal with CPC, all the relative expressions of detected immune barrier genes elevated first and then declined. Markedly higher relative expressions of *occludin*, *ZO1*, *MLCK*, *Claudin* 3, *Claudin 7*, and *Claudin 11* were observed in the CPC50 group (*p* < 0.05). In addition, except for the relative expressions of *ZO1*, the lowest relative expressions of detected immune barrier genes appeared in the CPC100 group, in which the relative expressions of *MLCK*, *Claudin3*, and *Claudin7* were significantly lower compared to the other group (*p* < 0.05).

**Figure 7:**
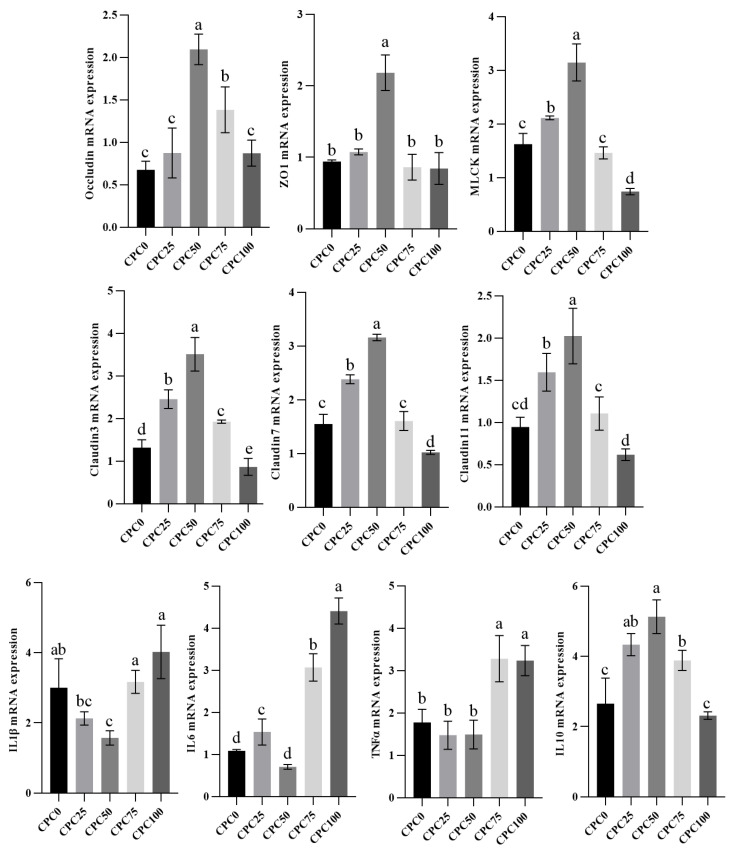
Intestinal relative expressions of immune barrier and cytokines genes of common carp fed diets with fish meal substituted by CPC. Values were presented as mean ± SE. Bars with different letters indicated significant differences (*p* < 0.05).

Regarding proinflammatory factors (*IL1β*, *IL6*, and *TNF-α*) in the intestine, the highest relative expressions were observed in the CPC100 group, while the lowest relative expressions appeared in the CPC50 group. Concretely, the CPC100 group possessed markedly higher *IL6* relative expressions than the other group, markedly higher *IL1β* relative expression than the CPC25 and CPC50 groups, and markedly higher *TNF-α* relative expression than the other groups except the CPC75 group (*p* < 0.05). On the contrary, the CPC100 group obtained markedly lower *IL10* (anti-inflammatory factor) relative expression in comparison to the other groups except the CPC0 group (*p* < 0.05). Noteworthy, the markedly highest *IL10* (anti-inflammatory factor) relative expression was observed in the CPC50 group (*p* < 0.05) (Figure 7).

To sum up, the optimal ratio of replacing fishmeal with CPC in common carp feed was estimated at 25–50%; however, the higher 75–100% replacement ratio would severely inhibit the growth, intestinal digestion, and immunity of common carp. Considering the results mentioned above, the control group (CPC0), the optimal replacement group (CPC50), and the excessive replacement group (CPC100) were selected to investigate the variation in intestinal histology and the intestinal microbiota composition.

### 3.8. Histological Analysis of the Midguts

**Figure 8:**
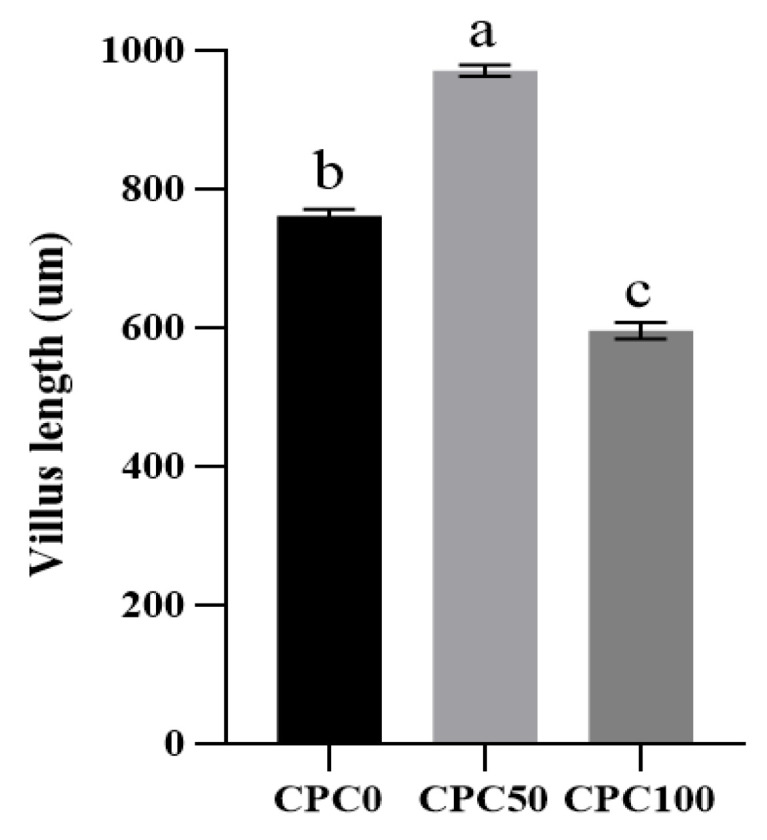
Intestinal villus height of common carp fed diets with fish meal substituted by CPC. Values were presented as mean ± SE. Bars with different letters indicated significant differences (*p* < 0.05).

**Figure 9:**
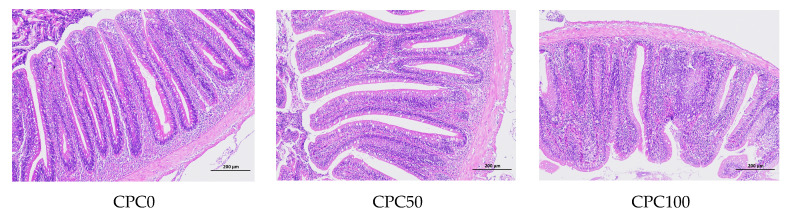
Intestinal histomorphology of common carp-fed diets with fish meal substituted by CPC.

Histological changes in the midgut specimens induced by the replacement of fishmeal with CPC are shown in Figure 8 and Figure 9. In terms of the villus height, the CPC50 group significantly elevated by approximately 27.43% compared to the CPC0 group (*p* < 0.05), whereas the CPC100 group significantly reduced by approximately 21.84% compared to the CPC0 group (*p* < 0.05). Moreover, the replacement of fishmeal with CPC caused the differentiation of the intestinal morphology of common carp. Firstly, although the intestines in the CPC0 and CPC50 groups exhibited neat structure and intact walls, specific differences in the state of the intestinal villi between the two groups were still observed. The CPC0 group presented the developed villi without fusion and shedding and relatively dense but low-height villi, while the CPC50 group showed a small amount of fusion and relatively sparse and higher height. Different from the previous two groups, the CPC100 group showed the worst intestinal morphology, in which the villi appear to be shed, heavily fused, blurred in outline, and lowest in height.

### 3.9. Intestinal Microbiota Analyses

The alpha diversity, containing the ACE index, CHAO index, SOBS index, shannon index, and simpson index, was often used to quantify the diversity and richness of each sample from the intestine. The coverage of the three groups was 99.72–99.83%, indicating that the sequencing amount was sufficient and could reflect the real situation of the intestinal microbiota of common carp. The current results indicated that there were no significant differences among the three groups in the five indices (Table 4). A Venn diagram derived from 16S rRNA gene sequencing was depicted to evaluate the predominant operational taxonomic units (OTUs) among the three groups.

**Figure 10:**
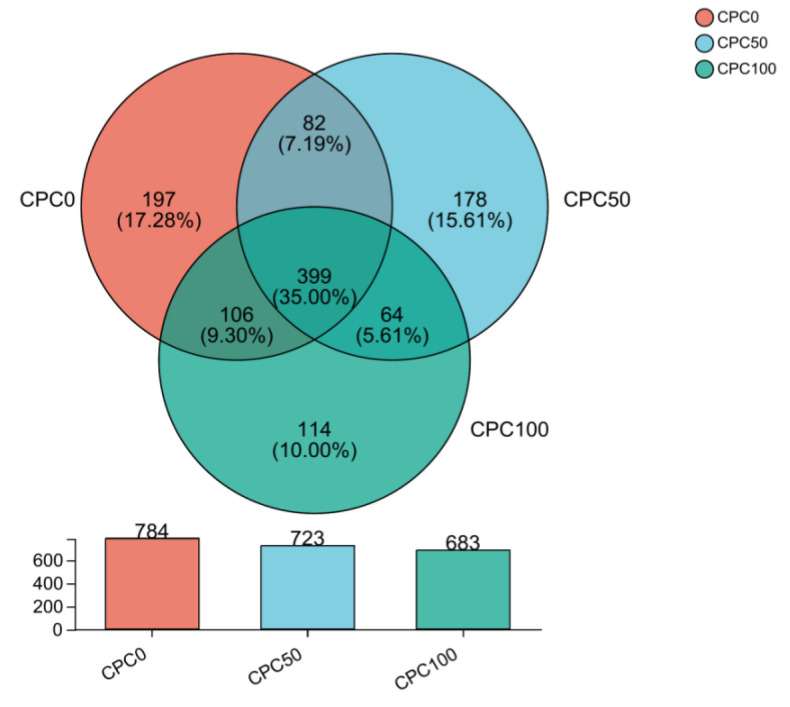
Venn diagram for the comparison of operational taxonomic units (out) among three groups.

There were a total of 1140 OTUs in the intestinal samples of the three groups, among which 399 were common OTUs, accounting for 35.00% of the total OTUs of the three groups. Specifically, the CPC0 group and CPC50 group jointly owned 82 OTUs, accounting for 10.46% of the total OTUs in the CPC0 group and 11.34% of the total OTUs in the CPC50 group; the CPC50 group and CPC100 group jointly owned 64 OTUs, accounting for 8.85% of the CPC50 group and 11.34% of the total OTUs in the CPC100 group; and the CPC100 group and CPC0 group jointly owned 106 OTUs, accounting for 15.52% of the CPC100 group and 13.52% of the total OTUs in the CPC0 group (Figure 10).

**Figure 11:**
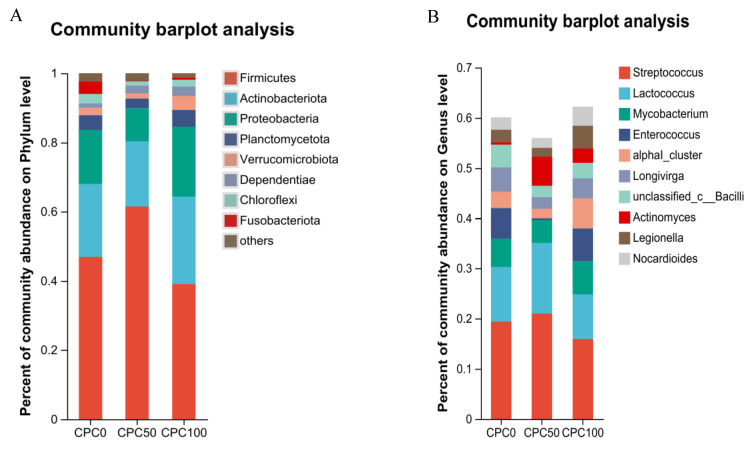
Intestinal microflora structure of each group at phylum (**A**) and genus (**B**) levels.

The microbiota of the intestine in the CPC0, CPC50, and CPC100 groups was investigated at the phylum and genus levels (Figure 10). At the phylum level, the dominant phylum (relative abundance > 5% at least in one sample) in three groups was Firmicutes (CPC0 = 46.93%, CPC50 = 61.49%, CPC100 = 39.02%), Actinobacteria (CPC0 = 21.04%, CPC50 = 18.82%, CPC100 = 25.27%), Proteobacteria (CPC0 = 15.76%, CPC50 = 9.59%, CPC100 = 20.29%), Planctomycetota (CPC0 = 4.27%, CPC50 = 2.81%, CPC100 = 4.76%), Verrucomicrobia (CPC0 = 2.08%, CPC50 = 1.46%, CPC100 = 4.06%), Dependentiae (CPC0 = 1.33%, CPC50 = 2.29%, CPC100 = 2.77%), Chloroflexi (CPC0 = 2.69%, CPC50 = 1.22%, CPC100 = 1.92%), Fusobacteriota (CPC0 = 3.55%, CPC50 = 0.09%, CPC100 = 0.57%), and others (CPC0 = 2.44%, CPC50 = 2.23%, CPC100 = 1.34%) (Figure 11A). The results showed that *Firmicutes*, *Actinobacteria*, *Proteobacteria*, and *Planctomycetota* comprised the top four predominant bacterial phylum in the common carp intestine among the three groups. In terms of specific composition, the relative abundance of *Firmicutes* was statistically increased in the CPC50 group, but the relative abundances of *Actinobacteria*, *Proteobacteria*, and *Planctomycetota* were statistically decreased compared to the CPC0 and CPC100 groups.

In combination with the comparison results of microbiota taxa at the genus level, the four primary genera (relative abundance > 5% at least in one sample) were *Streptococcus*, *Lactococcus*, *Mycobacterium*, and *Enterococcus* (Figure 11B). As Figure 11B illustrates, the relative abundances of *Streptococcus* and *Lactococcus* were statistically elevated in the CPC50 group, whereas the relative abundance of *Enterococcus* were statistically reduced in the CPC50 group compared to the CPC0 and CPC100 groups.

## 4. Discussion

### 4.1. Suitable Substitution of Fishmeal with CPC Contributes to the Growth-Promoting Effect

As a representative new source of plant proteins, CPC is derived from cottonseed with the separation of cotton shell and cotton kernel and the removal of gossypol and aflatoxins based on the “Liquid-liquid-solid” three-phase extraction method [29]. This not only reduces the amount of high-gossypol cottonseed meal but also strengthens the high-quality cottonseed protein utilization rate. A previous study found that 50% dietary fishmeal could be substituted by CPC without growth inhibition for pearl gentian groupers (*Epinephelus fuscoguttatus*♀ × *E. lanceolatus*♂) with an average initial weight of 11.97 ± 0.04 g [30]. Also, for juvenile hybrid groupers (*Epinephelus fuscoguttatus*♀ × *Epinephelus lanceolatus*♂) with an initial weight of 16.91 ± 0.02 g, replacing 60% fishmeal with CPC has no significant impact on the growth performance [31]. Similarly, Liu et al. [32] reported that no significant detrimental effects on growth performance or feed intake were observed in rainbow trout (*Oncorhynchus mykiss*) at the 75% substitution rate of dietary fishmeal by CPC. These findings surprisingly revealed the unpredictable feasibility of replacing fishmeal with CPC in carnivorous fish feed, which weakens the perception that carnivorous fish cannot effectively utilize plant protein efficiently. For a first attempt at fishmeal replacement with CPC on common carp, our study illustrated that dietary fishmeal could be substituted by CPC up to 46.47–47.72% based on the broken-line model analysis of MGR, SGR, and FCR against the dietary CPC replacement level. Concretely, the designed CPC50 group obtained superior MGR, SGR, FCR, and PER compared to the CPC-substitution groups, which implied that CPC has nutritional potential as a substitute for fishmeal in feed for omnivorous fish. As a support for our finding, a study of Nile tilapia (*Oreochromis niloticus*) showed that less than 30% soybean meal replaced by CPC did not lead to a significant decrease in growth condition [33]. There is also a consensus among the studies mentioned above that the significantly reduced growth performance in fish fed a diet with excessively high levels of CPC may be linked to poor blood biochemistry, influenced digestibility, weakened intestinal antioxidant and immune barriers, and disrupted intestine microbiota [15,18,31,32,33]. Therefore, we will focus on these aspects of the in-depth discussion.

The growth of fish is effectively regulated by the GH/IGF axis and protein synthesis ability to some extent. The GH/IGF axis helps regulate protein utilization and is involved in physiological processes such as cell growth, environmental adaptation, immune response, reproductive maturation, etc. [34]. The TOR pathway serves as a major sensitive pathway for dietary protein nutrition and regulates cell growth and metabolism [35]. Existing research has shown that a suitable replacement of fishmeal or soybean meal with novel protein sources could play a role in promoting growth by regulating the above two pathways [7,36]. In our study, the relative expression levels of genes involved in the GH/IGF axis and TOR pathway, such as GH, IGF1, PI3K, AKT, TOR, S6K, and 4ebp1, were up-regulated first and then down-regulated with an increasing proportion of CPC substitutions, somewhat analogous to the variation tendency of growth parameters. The above results could demonstrate that the appropriate substitution level of CPC could elevate the protein synthesis capacity by activating the GH/IGF axis and the TOR pathway, thereby promoting growth performance. Similar findings were also captured in a study of swimming crabs (*Portunus trituberculatus*) [37]. In addition, it cannot be denied that the immoderate substitution level of CPC would restrain the functionality of the GH/IGF axis and TOR pathway, which might be attributed to the lower limit of gossypol tolerance [38] or the imbalance of amino acids in diets (particularly arginine and leucine) [39,40] induced by the overmuch CPC addition. The specific regulatory mechanisms involved need to be further explored.

### 4.2. Suitable Substitution of Fishmeal with CPC Improves Serum Biochemical Composition

The analysis of serum index is a critical assessment tool for physiological, pathological, and nutritional evaluation that can effectively reflect the metabolism, nutritional status, and disease status of fish [41]. In our study, we mainly investigated the serum biochemical indexes, which reflect the protein metabolism and lipid metabolism of fish. TP content in serum can reflect the level of protein metabolism in aquatic animals, and BUN from protein catabolism will increase with a poor state of absorption and utilization of protein in vivo [41]. The present research showed that common carp in the CPC50 group possessed higher TP contents and lower BUN contents in comparison to the control group and excess substitution group (CPC100), suggesting that 50% fishmeal replacement with CPC for common carp can improve the level of protein metabolism, accelerate the transport of metabolites, and efficiently absorb and utilize proteins. An analogous variation was also observed in the study of *Trachinotus ovatus* [42] and our previous study [7]. Besides, GOT and GPT in the liver cells can catalyze various reactions to synthesize amino acids and provide sufficient substrates for protein synthesis. When the hepatocytes undergo inflammatory and toxic reactions, the impaired hepatocytes release large amounts of hepatic aminotransferases into the bloodstream, resulting in elevated serum aminotransferase levels. This study found that fishmeal replaced by CPC in common carp diets elevated the level of serum transaminase to some extent, which was consistent with the result of Zheng et al. [43]. This may be due to the high proportion of CPC replacement, which results in changes in the amino acid composition of the feed. Serum lipid composition reflects the ability of liver lipid metabolism and lipid absorption to a certain extent [44]. This study revealed that common carp in the CPC group acquired the highest HDL-C content, and the lowest TG and HDL-C contents suggested that suitable CPC replacement may promote lipid metabolism. Similar situations were pointed out in the studies of fishmeal replacement by CPC in largemouth bass [45] and by cottonseed meal in grass carp [46]. Meanwhile, it should not be ignored that the inordinate fishmeal replacement by CPC may result in cell dysfunction and metabolic disorders, as reflected in the excessively low contents of TP and HDL-C and the superfluous contents of TCHO, TG, and LDL-C.

In combination with the available findings, a consensus has been established that for stomachless fish (grass carp, common carp, etc.), intestinal health has become a key factor in promoting the growth performance of fish with the continuous updating of dietary protein sources [7,47]. As a consequence, our study investigated in detail the intestinal response to the replacement of fishmeal with CPC depending on state presentations of digestion, morphology, antioxidation, tight junction, and microbiota.

### 4.3. Suitable Substitution of Fishmeal with CPC Elevates Intestinal Digestive and Absorptive Ability

Among all intestinal functions, digestion and nutrient absorption are principal in facilitating fish growth. In the first place, the morphological basis of the intestine determines the function of the intestine [48]. The villus height is a positive indicator of intestinal digestion and nutrient absorption, which is attributed to their ability to maintain an intact and healthy mucosal epithelium [49]. This study demonstrates that 50% fishmeal replacement with CPC significantly increased intestinal villus height, whereas 100% fishmeal replacement with CPC significantly restrained the villus height, implying that suitable CPC substitution levels (50%) for common carp significantly improved intestinal morphology. A study on the large yellow croaker (*Larimichthys crocea*) has also concluded that ≥60% dietary CPC replacement levels could alter the structure of the intestine and therefore damage intestinal absorptive ability [50]. The role of CPC in promoting intestinal digestion and nutrient absorption is embodied not only in intestine morphology but also in the activities of major digestive enzymes. Protease, lipase, and amylase are important digestive enzymes to digest dietary proteins, lipids, and starch. The current study showed that, compared to the control group and other replacement groups, 50% fishmeal replacement with CPC markedly elevated the protease and lipase activities in the whole intestine and the amylase activity of the foregut and midgut. This variation tendency is different from the previous perception that fish intestinal digestive enzyme activity was strongly inhibited by plant protein sources replacing fishmeal in the diet [51,52]. Regrading to fishmeal replacement with CPC, Tian et al. [50] pointed out that the digestive enzyme activities (alpha-amylase, lipase, and trypsin) in large yellow croakers decreased linearly with increasing dietary fishmeal substitution levels by CPC. This disagreement may be mainly due to the differentiation of major digestive organs between different fish (intestine for common carp, stomach for large yellow croaker). But nonetheless, when CPC substitution levels surpassed 50% and reached 75–100%, the intestinal digestive enzyme activities were obviously restricted for common carp. A similar result was observed in the study on the sturgeon (*Acipenser schrenckii*) [53]. These current findings demonstrated that the suitable fishmeal replacement level by CPC produced positive impacts on the digestive and absorptive capacity of common carp, which seems to have became one of the reasons for promoting feed utilization.

### 4.4. Suitable Substitution of Fishmeal with CPC Remits the Weakening Effect of Reducing Fiashmeal on the Intestinal Antioxidant Capacity and Immune Function

For aquatic animals, the destruction of oxidative homeostasis will result in oxidative stress and further impairment of tissue function [54]. Particularly within the intestine, its antioxidant capacity serves to ensure intestinal structural integrity and functional stability [55], which are affected by the breeding conditions represented by the composition of feed materials [56,57]. To date, there has been ample evidence that high levels of plant-based protein sources replacing fish meal cause oxidative damage [58,59]. As a support, this current study showed that ≥75% fishmeal replacement with CPC significantly increased intestinal MDA contents, which act as biomarkers of oxidative damage [60]. To maintain redox homeostasis, cells have antioxidant systems that include antioxidant enzymes (SOD, CAT, and GSH-PX) and antioxidant substances (GSH) [61]. In the present study, reductions of antioxidant enzymes (SOD, CAT, and GSH-PX) were predictably observed in common carp fed the CPC75 and CPC100 diets. However, it was heartening that common carp fed the CPC50 diets captured significantly higher activities of SOD, CAT, GSH-PX, and GSH and lower MDA contents. The above description revealed that common carp intestinal antioxidant capacity exhibited a tendency to rise first and then decrease when the substitution level of fish meal with CPC gradually increased, which aligns with the previous study with hybrid groupers. The activity levels of the above antioxidant enzymes reflect the ability of corresponding genes to regulate antioxidation. As expected, substituting fishmeal with CPC up to 50% triggered an up-regulation in relative expressions of *Cu/Zn-SOD*, *CAT*, and *GSH-Px* compared with the control group and other substitution groups, whereas subsequent 75% and 100% substitution levels inhibited the up-regulation of antioxidant genes. The Keap1 (Kelch-like ECH-associated protein 1)-Nrf2 (NF-E2-related factor 2) signaling pathway exerts a moderating role in the transcription of antioxidant genes in the process of defending against oxidative stress [60,62]. To cope with oxidative stress, Nrf2, as a crucial transcription factor, combines with the antioxidant reaction element ARE to initiate the expression of a series of downstream protective genes, whereas Keap1 plays a repressive role in Nrf2 activation [63]. The current results indicated that substituting fishmeal with CPC up to 50% aroused a decrease in relative expression of *Keap1* and an increment in relative expression of *Nrf2*, and the higher substitution levels reversed this variation trend. Hence, the improved antioxidant ability of common carp under a 50% CPC substitution level may be partly ascribed to the heightened effective regulation of the Keap1-Nrf2 signaling pathway in the intestine. Such an effect on common carp has been found in our previous research on the replacement of soybean meal with fermented peanut meal [7].

An imbalance in oxidative status usually causes intestinal inflammation, in which pro- and anti-inflammatory cytokines have been viewed as markers in evaluation indicators [64]. In this study, marked increments in the gene expressions of pro-inflammatory cytokines (i.e., *IL1β*, *IL6*, and *TNF-α*) and reductions in the gene expressions of anti-inflammatory cytokines (i.e., *IL10*) were observed in the CPC50 group, which indicated that reinforcement in antioxidant capacity (including enzyme activities and gene expressions) by CPC replacing 50% fishmeal could be conductive to weakening intestinal inflammation induced by an increase in plant protein sources. However, when the CPC replacement ratio exceeds 50%, the intestine will suffer from inflammation. This result is consistent with previous cognition that the extensive use of plant protein sources in feed has negative effects on the intestinal immune function of fish, thereby causing food-borne inflammation [32,65,66]. As introduced by Yin et al. [62], substituting fishmeal with CPC in hybrid grouper feed beyond 36% significantly upregulated the mRNA levels of pro-inflammatory cytokines and down-regulated the mRNA levels of anti-inflammatory cytokines hepcidin, thereby aggravating intestinal inflammation. This may be potentially associated with the excess of anti-nutritional factors caused by high levels of substitution.

### 4.5. Suitable Substitution of Fishmeal with CPC Enhances the Intestinal Mechanical Barrier and Biological Barrier

The intestinal mechanical barrier is an important defense line against the invasion of pathogens, and is mainly composed of epithelial cells and an intercellular tight junction. The permeability of the intestinal tight junctions determines the barrier function of the entire intestinal epithelium [67]. Tight junction proteins, including transmembrane proteins (occludin, claudins) and peripheral cytoplasmic proteins (ZO), form a narrow band structure from the outside to the inside, which prevents toxic substances from entering the surrounding tissues by sealing the cell space [68]. In this study, the gene expressions of tight junction proteins elevated first and then decreased with increasing CPC replacement ratio, and the tipping point is 50%. Unlike our study, studies on hybrid grouper [62] and largemouth bass [69] found that replacement of fishmeal with CPC damaged intestinal structure and tight junctions, and this destructiveness is marked significantly when the substitution ratio is excessively high (75% for hybrid grouper and 60% for largemouth bass). This may be due to that, compared to largemouth bass, common carp possess a stronger adaptability to plant-based proteins.

The intestinal biological barrier mainly consists of normal intestinal microbiota, which can promote the proliferation of intestinal epithelial cells and strengthen the immune function of fish [70] and is tightly coupled with dietary nutrient composition [71]. In our study, the control group CPC0, the optimal substitution group CPC50, and the excess substitution group CPC100 were chosen to evaluate the intestinal microbiota composition according to the correlation results of growth and intestinal immune indicators. For one thing, dietary fishmeal replacement with CPC reduced the diversity of microbiota composition in the intestine. For another thing, dietary fishmeal replacement with CPC altered the proportion of major microbiota. From the perspective of the phylum, common carp fed the CPC50 diets had a higher proportion of *Firmicutes* abundance (61.49%) in comparison to those fed the CPC0 (46.93%) and CPC100 (39.02%) diets. The variation resembles the studies on carnivorous fish species, including golden pompan [15], rainbow trout [32], and largemouth bass [72]. But different from them, the abundance proportions of both *Actinobacteria* and *Proteobacteria* with pathogenicity were declined in the CPC50 group and elevated in the CPC100 group. It might prove, once again, that common carp can make better use of plant protein sources than carnivorous fish species. In addition, the analysis of the genus level indicated that *Streptococcus*, *Lactococcus*, *Mycobacterium*, and *Enterococcus*, which belong to Firmicutes, are the four most dominant bacterial genera. Among these, *Lactococcus* can regulate the immune response of the animal body, resist pathogenic bacteria, and improve the intestinal morphology and structure [73]. Enterococcus is one of the most important infectious pathogens in Gram-positive bacteria, second only to *Staphylococcus*, which can cause pathological changes in nutrient deficiency or a high alkaline environment, leading to infection and disease [74]. In the current study, a higher proportion of *Lactococcus* abundance was observed in the CPC50 group compared to the CPC0 and CPC100 groups. This seems to suggest that when the replacement ratio is 50%, the main bacteria in the intestine are beneficial to intestinal digestion and immunity. Inversely, The higher proportion of *Enterococcus* abundance was observed in the CPC100 group compared to the CPC0 and CPC50 groups, suggesting that excessive Enterococcus in the CPC100 group may be an induction factor in reducing the immune capacity of the intestine. Taken together, CPC replacing 50% fishmeal could enhance the intestinal mechanical barrier by up-regulating the gene expressions of tight junction proteins and heighten the intestinal biological barrier by increasing the probiotics and decreasing the harmful bacteria.

## 5. Conclusions

In conclusion, the results of the present study indicated that 50% fishmeal replacement with CPC in common carp feed was conducive to growth performance via activating the GH/IGF axis and TOR pathway, intestinal digestive ability via improving intestinal morphology and digestive enzyme activities, intestinal antioxidant capacity via evoking the Nrf2/Keap1 signal pathway, and intestinal immune response via enhancing the mechanical barrier and biological barrier. But excessive substitution levels (75% and 100%) would compromise growth performance, intestinal antioxidant capacity, and immune function. Based on the broken-line regression analysis of MGR, PER, and FCR against the dietary CPC replacement levels, the optimal replacement level of fish meal with CPC was estimated to be 46.47%, 47.72%, and 46.43% for juvenile common carp, respectively. This study will lay a solid foundation for CPC as an alternative to fishmeal in aquafeeds.

## Figures and Tables

**Table 1:** Ingredients and proximate compositions of experimental diets (g/kg dry matter).

Ingredients	CPC0	CPC25	CPC50	CPC75	CPC100
Soybean meal ^a^	260	260	260	260	260
Wheat middlings ^a^	417	415.5	414.3	413	411.4
Fishmeal ^a^	100	75	50	25	0
Soybean protein concentrate ^a^	50	50	50	50	50
Rapeseed meal ^a^	100	100	100	100	100
Cottonseed protein concentrate ^a^	0	25	50	75	100
Fish oil	20	20.5	20.8	21.1	21.6
Soybean oil	20	20.5	20.8	21.1	21.6
Vitamin premix ^b^	3	3	3	3	3
Choline chloride	3	3	3	3	3
Dicalcium phosphate	20	20	20	20	20
Trace mineral premix ^c^	2	2	2	2	2
Lysine	3	3.4	3.9	4.4	4.9
Methionine	2	2.1	2.2	2.4	2.5
Total	1000.00	1000.00	1000.00	1000.00	1000.00
Moisture	104.7	104.6	105.0	105.0	104.9
Crude protein	305.7	305.5	305.4	305.2	305.0
Crude lipid	57.8	58.0	57.9	57.7	57.9
Crude ash	57.0	57.7	58.6	59.4	60.1
Crude fibre	58.7	57.0	55.3	53.6	51.8
NFE ^d^	416.0	417.0	417.9	419.2	420.3
Crude energy (kJ/g) ^e^	16.65	16.68	16.68	16.69	16.71

^a^ Fish meal: crude protein 670 g/kg dry matter, crude lipid 48.5 g/kg dry matter; Soybean meal: crude protein 440 g/kg dry matter, crude lipid 15 g/kg dry matter; Wheat middling: crude protein 166 g/kg% dry matter, crude lipid 12 g/kg% dry matter; Soybean protein concentrate: crude protein 630 g/kg dry matter, crude lipid 5 g/kg dry matter; Rapeseed meal: crude protein 380 g/kg dry matter, crude lipid 38 g/kg dry matter. ^b^ Vitamin mixture (g/kg mixture) supplied by Guangdong Hyint Biotechnology Group Co., Ltd., Guangzhou, China: vitamin A(VA) 8000 IU, vitamin C (VC) 100 mg, vitamin D3 (VD3) 3000 IU, vitamin E (VE) 60 mg, vitamin K3 (VK3) 5 mg, vitamin B1 (VB1) 15 mg, vitamin B2 (VB2) 30 mg, vitamin B6 (VB6) 15 mg, vitamin B12 (VB12) 0.5 mg. ^c^ Trace mineral mixture (mg/g mixture) supplied by Guangdong Hyint Biotechnology Group Co., Ltd.: nicotinamide 175 mg, d-biotin 2.5 mg, inositol 1000 mg, folic acid 5 mg, pantothenic acid 50 mg, zinc (Zn) 60 mg, copper (Cu) 3 mg, iron (Fe) 25 mg, manganese (Mn) 15 mg, iodine (I) 0.6 mg, and magnesium (Mg) 0.7 mg. ^d^ NFE = nitrogen-free extract. Estimated by the formula 1000 − (water + crude protein + crude fibre + ashes + crude lipid). ^e^ Calculated using the mean values for carbohydrates (17.2 kJ/g), proteins (23.6 kJ/g), and lipids (39.5 kJ/g).

**Table 2:** Sequence of the primers used for this study.

Gene Name	Primer Sequence (5′-3′)	Serial Number
Growth axis		
*GH*	F: ATCTTCCCTCTGTCTTTCTGC R: AAGTCGGCCAGCTTCTCA	M27000.1
*IGF1*	F: AGACAGCCCAAGGACAGCA R: TACAGTGGAGCACATCTCTGGAA	D83272.1
Protein synthesis		
*PI3K*	F:AAGACCTTCCTCATCACGAC R:CCTTCCACTACAACACTGCA	XM_042758409.1
*AKT*	F:GGTGTGTTCAAGTTCACCGTCT R: TCCTCACCCAGCTCTCCA	XM_042722896.1
*TOR*	F:CCACAACGCAGCCAACAA R:GCCACAGAATAGCAACCCT	AF119837.1
*S6K*	F:GCCAATCTCAGCGTTCTCAAC R:CTGCCTAACATCATCCTCCTT	EF373664.1
Antioxidation		
*Nrf2*	F: TTCCCGCTGGTTTACCTTAC R: CGTTTCTTCTGCTTGTCTTT	JX462955
*Keap1*	F: CTACAACCCCGAGAGACGA R: GGAGGAGATGAAGCTCCAGAC	JX470752
*CuMnSOD*	F: TGGCGAAGAAGGCTGTTTGT R: TTCACTGGAGACCCGTCACT	JF342355
*CAT*	F: CTGGAAGTGGAATCCGTTTG R: CGACCTCAGCGAAATAGTTG	JF411604
*GPX1a*	F: GTGACGACTCTGTGTCCTTG R: AACCTTCTGCTGTATTCTCTTGA	JF411605
*GPX1b*	F: TATGTCCGTCCTGGCAATGG R: ATCGCTGGGAATGGAAGTT	JF411606
Immune barrier		
*occludin*	F: ATCGGTTCAGTACAATCAGG R: GACAATGAAGCCCATAACAA	KF975606
*ZO-1*	F:GCCTGCCTACACTCAACCACAAC R:CTGCTTCGGCTGGAGGAGGAG	KY290394.1
*Claudin3*	F: GCACCAACTGTATCGAGGATG R: GGTTGTAGAAGTCCCGAATGG	JQ767157
*Claudin7*	F: CTTCTATAACCCCTTCACACCAG R: ACATGCCTCCACCCATTATG	JQ767155
*Claudin11*	F: TCGGAAGTGAACCAGAAAGC R: GAAGCCAAAGGACATCAAGC	JQ767158
*MLCK*	F: AGCAGTGTGGGCATCAACCT R: CTCCAGCAGGGTCATGATGAG	XM_019076433.1
Cytokines		
*IL-1β*	F: AACTTCACACTTGAGGAT R: GACAGAACAATAACAACAAC	KC008576
*IL-6*	F: TAGGTTAATGAGCAAGAGGA R: AGAGACTGTTGATACTGGAA	AY102633.1
*IL-8*	F:AAACTGAGAGTCGACGCATTG R:TTTTCAATGACCTTCTTAACCCAG	EU011243.1
*IL-10*	F: GCCAGCATAAAGAACTCG R: CCAAATACTGCTCGATGT	JX524550.1
*TNF-α*	F: AAGTCTCAGAACAATCAGGAA R: TGCCTTGGAAGTGACATT	AJ311800
Internal reference		
*β-actin*	F: GGCAGGTCATCACCATCGG R: TTGGCATACAGGTCTTTACGG	M24113.1

Note: growth hormone, GH; insulin growth factor 1, IGF1; phosphatidylinositol-3-kinase, PI3K; protein kinase B, AKT; target of rapamycin, TOR; S6 kinase, S6K; nuclear factor (erythroid-derived 2)-like 2, Nrf2; Kelch-like epichlorohydrin-related protein, Kelch-l; catalase, CAT; glutathione peroxidase 1a, GPx1; zonula occluden 1, ZO-1; myosin light chain kinase, MLCK; interleukin-1β, IL-1β; interleukin-6, IL-6; interleukin-8, IL-8; interleukin-10, IL-10; tumor necrosis factor α, TNF-α.

**Table 3:** Biochemical components of the serum of common carp-fed diets with fish meal substituted by CPC.

Items	CPC0	CPC25	CPC50	CPC75	CPC100
TP (g/L)	27.00 ± 1.98 ^c^	42.62 ± 2.41 ^ab^	48.53 ± 0.28 ^a^	42.68 ± 0.89 ^ab^	40.04 ± 3.31 ^b^
BUN (mmol/L)	5.78 ± 0.14 ^a^	3.75 ± 0.31 ^c^	3.53 ± 0.33 ^c^	4.69 ± 0.23 ^b^	5.99 ± 0.30 ^a^
ALT (U/gprot)	19.28 ± 0.54 ^c^	19.01 ± 0.39 ^c^	20.45 ± 0.18 ^ab^	21.27 ± 0.18 ^a^	19.64 ± 0.18 ^bc^
AST (U/gprot)	63.55 ± 2.73 ^c^	69.94 ± 2.31 ^c^	81.8 ± 2.93 ^b^	77.8 ± 2.58 ^b^	109.57 ± 0.72 ^a^
TC (mmol/L)	6.02 ± 0.10 ^d^	7.57 ± 0.12 ^a^	7.05 ± 0.02 ^b^	5.76 ± 0.76 ^d^	6.46 ± 0.17 ^c^
TG (mmol/L)	7.12 ± 0.31 ^a^	5.46 ± 0.65 ^b^	5.13 ± 0.22 ^b^	7.07 ± 0.43 ^a^	7.83 ± 0.34 ^a^
HDL-C (mmol/L)	0.57 ± 0.23 ^e^	1.15 ± 0.23 ^b^	1.44 ± 0.01 ^a^	0.91 ± 0.24 ^c^	0.65 ± 0.22 ^d^
LDL-C (mmol/L)	4.9 ± 0.03 ^b^	4.49 ± 0.52 ^b^	4.34 ± 0.2 ^b^	5.04 ± 0.39 ^b^	7.9 ± 0.98 ^a^

Different letters within the same column are significantly different (Duncan’s test, *p* < 0.05). The data were expressed as the mean ± SE.

**Table 4:** Alpha diversity index of samples.

Groups	ACE	CHAO	SOBS	SHANNON	SIMPSON	COVERAGE (%)
CPC0	472.83 ± 42.22	479.58 ± 40.79	426.50 ± 50.14	3.59 ± 0.45	0.12 ± 0.06	99.76 ± 0.01
CPC50	459.59 ± 91.13	463.59 ± 91.52	426.83 ± 87.77	3.91 ± 0.87	0.09 ± 0.08	99.81 ± 0.02
CPC100	460.82 ± 57.07	462.3 ± 60.32	402.83 ± 56.27	3.52 ± 0.48	0.11 ± 0.05	99.74 ± 0.02

## Data Availability

The data presented in this study are available on request from the corresponding author.
